# Automatic Pretreatment of Dispersive Liquid Liquid Microextraction Based on Immunomagnetic Beads Coupled with UPLC-FLD for the Determination of Zearalenone in Corn Oils

**DOI:** 10.3390/toxins15050337

**Published:** 2023-05-15

**Authors:** Baoxia Ni, Jin Ye, Zhihong Xuan, Li Li, Xiangrui Wen, Zongwang Li, Hongmei Liu, Songxue Wang

**Affiliations:** 1 Academy of National Food and Strategic Reserves Administration, No. 11 Baiwanzhuang Street, Beijing 100037, China; nbx@ags.ac.cn (B.N.); yj@ags.ac.cn (J.Y.); xzh@ags.ac.cn (Z.X.); ll@ags.ac.cn (L.L.); qch120731@163.com (X.W.); lzw18735877886@163.com (Z.L.); wsx@ags.ac.cn (S.W.); 2 College of Food Science and Engineering, Central South University of Forestry and Technology, Changsha 410004, China; 3 School of Health Science and Engineering, University of Shanghai for Science and Technology, Shanghai 200093, China

**Keywords:** immunomagnetic beads, dispersive liquid liquid microextraction, pretreatment, zearalenone, corn oils, UPLC-FLD

## Abstract

Sample pretreatment is a vital step in the detection of mycotoxins, and traditional pretreatment methods are time-consuming, labor-intensive and generate much organic waste liquid. In this work, an automatic, high-throughput and environmentally friendly pretreatment method is proposed. Immunomagnetic beads technology and dispersive liquid–liquid microextraction technology are combined, and the zearalenone in corn oils is directly purified and concentrated under the solubilization effects of surfactant. The proposed pretreatment method allows for the batch pretreatment of samples without pre-extraction using organic reagents, and almost no organic waste liquid is produced. Coupled with UPLC-FLD, an effective and accurate quantitative detection method for zearalenone is established. The recovery of spiked zearalenone in corn oils at different concentrations ranges from 85.7 to 89.0%, and the relative standard deviation is below 2.9%. The proposed pretreatment method overcomes the shortcomings of traditional pretreatment methods and has broad application prospects.

## 1. Introduction

Zearalenone (ZEN) is mainly produced by *Fusarium* species, which has estrogen-like activity and could improve the level of female hormones in animals and cause reproductive abnormalities. It also has cytotoxicity, immunotoxicity and hepatotoxicity, seriously endangering human and animal health [1,2]. The limit of ZEN in wheat, corn and their flour products is 60 μg/kg in China [3]. The detection rate of ZEN in grain crops, especially corn, is very high, and corn is the basic raw material of corn oil, so the pollution of ZEN in corn oil cannot be ignored [4]. Corn oil is a type of edible oils that residents consume daily. The European Union has set a limit of 200 μg/kg for ZEN in refined corn oil [5]. Therefore, it is necessary to develop and establish an efficient and accurate detection method for ZEN in corn oil. At present, the main methods for the determination of ZEN in food are enzyme-linked immunosorbent assay (ELISA) [6], colloidal gold immunochromatography method [7], liquid chromatography [8,9], etc. Because of its high accuracy and precision, liquid chromatography is the most commonly applied method of quantitative detection and is usually used in combination with pretreatment methods.

The corn oil matrix is complex and contains various potential interferences. A large number of triglycerides could be removed through sample pretreatment to reduce matrix effects, and target toxins could be enriched at the same time. Therefore, sample pretreatment is especially important for ZEN detection and analysis in corn oils [10]. The conventional pretreatment methods generally include extraction and purification processes. The extraction process generally requires a certain volume of organic reagents (methanol or acetonitrile) and includes steps such as long-term vortex and centrifugation. The common purification methods include solid-phase extraction column, immunoaffinity column (IAC), and the quick, easy, check, effective, rugged, and safe (QuEChERS) method [11,12,13,14]. The above pretreatment methods are not only complex, time-consuming and labor-intensive, but also generate much organic waste liquid, resulting in environmental pollution. Therefore, developing an efficient, environmentally friendly, and extraction purification integrated pretreatment method is of great significance for improving detection efficiency.

The immunomagnetic beads (IMBs) purification method has received extensive research in recent years due to its advantages of a simple operation, fast separation and strong specificity [15,16]. IMBs possess the advantages of a uniform particle size, large specific surface area, and easy surface modification. Utilizing the specific affinity of antibody on IMBs, the target toxin is identified and enriched from the sample, and then obtained through steps such as adsorption, magnetic separation, and elution. Wang et al. [17] established an ELISA based on IMBs for the detection of fumarotoxin B_1_ in corn. Compared with the conventional ELISA method, this method not only has higher sensitivity but also simpler operation steps. Liu et al. [18] applied IMBs as a purification method and combined it with UPLC to detect aflatoxin B_1_ in vegetable oil, which has the advantages of being fast and simple, with a high sensitivity and good accuracy. Our research group recently has conducted a series of studies on the pretreatment based on IMBs, achieving the rapid and automatic purification of grain and edible oil samples [19,20]. Although the IMBs-based method is convenient and fast in operation, the pretreatment process still requires many organic reagents to extract the target toxin in advance, especially regarding the weak polarity of ZEN molecules. Achieving non-organic reagent extraction for non-polar or weakly polar targets such as ZEN is very challenging.

Liquid-phase microextraction (LPME) technology is a convenient, efficient, and environmentally friendly pretreatment technology that applies a little liquid as an extractant to extract and separate target substances, overcoming the disadvantages of traditional methods [21,22]. Dispersive liquid–liquid microextraction (DLLME) is a type of LPME technology that has developed rapidly in recent years [23,24]. DLLME applies dispersants to improve the dispersibility of organic extractants in the aqueous phase, and could utilize auxiliary tools to reduce the dosage of organic reagents [25]. Galucha et al. [26] applied DLLME pretreatment technology combined with UHPLC-MS/MS to determine acrylamide in coffee. The pretreatment process used a little organic extraction solution and achieved good results. Farajzadeh et al. [27] developed a new, simple and efficient method based on evaporation of the sedimented organic phase obtained from DLLME for the analysis of multiclass pesticide residues from some fruit juice samples. Ju et al. [28] applied deep eutectic solvent as the extractant, and the developed DLLME method displayed satisfactory extraction efficiency for the three neonicotinoid pesticides in edible oils, and coupled this with HPLC for the detection. However, this technology is only suitable for several compounds or a class of compounds, and the sample matrix is relatively limited and cannot meet the requirements of high throughput and automatic pretreatment.

Herein, we combine IMBs technology with DLLME technology, assisting the mycotoxin automatic purification instrument developed by our group in the development of an immunomagnetic beads coupled with dispersive liquid liquid microextraction (IMBs-DLLME) pretreatment method for ZEN. Coupled with the Ultra-Performance Liquid Chromatography Fluorescence Detector (UPLC-FLD), an efficient, sensitive, and accurate quantitative detection and analysis method is established. This IMBs-DLLME method not only effectively shortens the pretreatment time and improves efficiency, but also avoids the influence of human factors on experimental results, and improves the accuracy, convenience, and repeatability of detection. In addition, the proposed pretreatment method realizes the non-organic reagent extraction of weakly polar ZEN, eliminates the step of pre-extraction with organic reagents, and greatly reduces the environmental pollution caused by the organic waste liquid generated in the pre-treatment process. The IMBs-DLLME pretreatment method coupled with UPLC-FLD meets the accuracy and sensitivity detection requirements of ZEN in corn oils.

## 2. Results and Discussion

### 2.1. Establishment of Analysis Method

**Figure 1:**
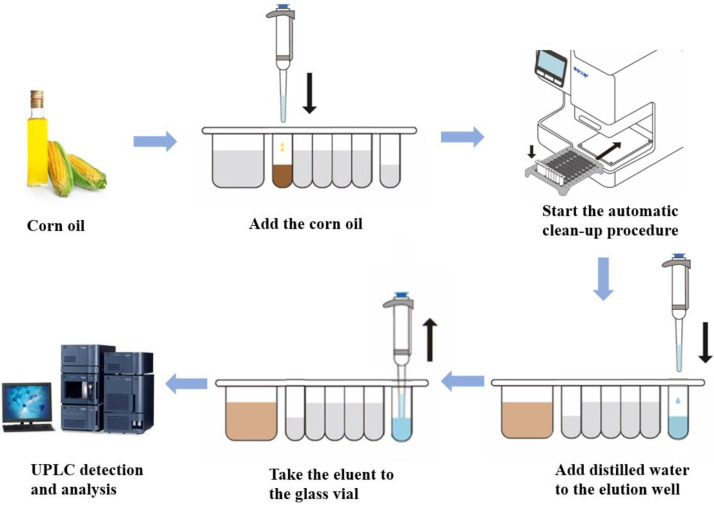
Schematic diagram of the proposed method.

The method of automatic IMBs-DLLME pretreatment with UPLC-FLD detection of ZEN in corn oils was established, and the schematic diagram is shown in Figure 1. The entire operation process was as follows: First, 0.25 mL of corn oil was added to the reaction well of the reagent kit, and then the kit was placed in the mycotoxin automatic purification instrument and the automatic purification process was initiated. Under the action of magnetic stick, the enrichment, washing and elution steps were respectively performed, and finally UPLC detection was performed. ZEN was enriched from the sample through specific binding of the ZEN antibody to IMBs, and interference impurities were removed through washing steps. Then, 0.5 mL organic reagent was used to disrupt the spatial structure of the antibodies on the IMBs, allowing ZEN to be released from the IMBs and subjected to detection. Three key factors, the reaction solution, incubation time and washing solution, were optimized, and the mechanism of the enrichment effects was explored.

#### 2.1.1. Optimization of Reaction Solutions

**Figure 2:**
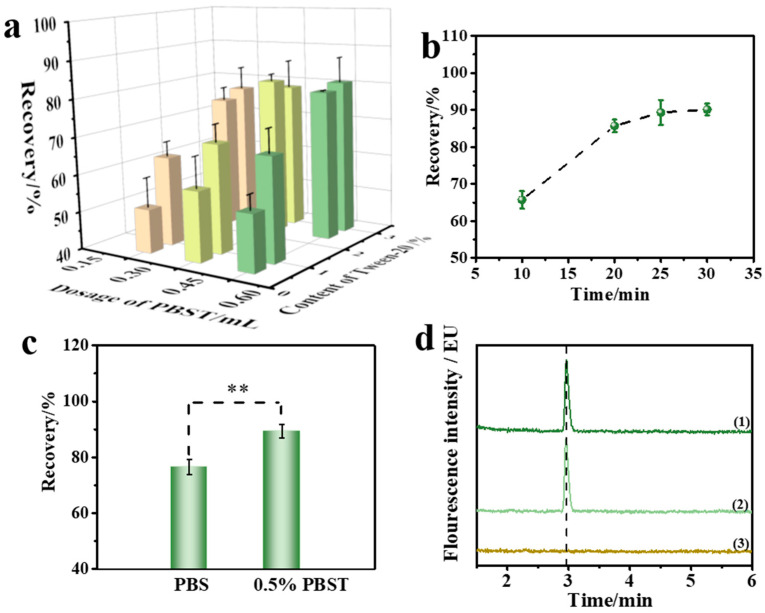
(**a**) Optimization of reaction solutions by checkerboard method. The effect of (**b**) incubation time; error bars represent the standard deviation (SD). (**c**) Washing solutions on recovery. ** represents significant difference. (**d**) UPLC chromatograms: (1) certified reference corn oils, (2) ZEN reference solutions, (3) blank solutions.

The IMBs solution, reaction solution (phosphate-buffered saline (PBS) solution containing Tween 20) and quality control materials of corn oil (ZEN concentration of 320 μg/kg) were added to the reaction well of the reagent kit. In the static state, the aqueous phase containing IMBs and reaction solution was in the lower layer, and the corn oil containing the target toxin in the upper layer, forming a water–oil layered state. Assisted by the mycotoxin automatic purification instrument, the water and oil phases formed a fine oil-in-water emulsion system during the vibration process of the magnetic stick. Due to the weak polarity of ZEN molecules, the solubility in aqueous solution was almost to vanishing point. By utilizing the solubilization effect of surfactants, an appropriate dosage of Tween 20 was added to the PBS solution to form the reaction solution and improved the solubility of ZEN in the reaction solution. Therefore, the dosage of the reaction solution and the concentration of surfactant were crucial and the checkerboard method was adopted to optimize the two key factors. The results are shown in Figure 2a. When the concentration of Tween 20 was 2.5% and the dosage of reaction solution was 0.45 mL, the recovery rate was the highest, reaching 81.3%. Therefore, 0.45 mL 2.5% PBST solution was selected as the optimal reaction solution.

#### 2.1.2. Optimization of Incubation Time

The incubation time between ZEN antibody (anti-ZEN) IMBs and ZEN in corn oil was another key factor affecting the enrichment efficiency. A sufficient incubation time was required to ensure the specific binding of the antibody on IMBs to ZEN, but it was also necessary to minimize the incubation time to improve the pretreatment efficiency. This experiment investigated the incubation time between IMBs and the quality control materials of corn oil. The IMBs, an appropriate volume of reaction solution, and corn oil (ZEN concentration of 320 μg/kg) were mixed, and incubation time (10, 20, 25, and 30 min) was investigated to evaluate the specific binding efficiency. As shown in Figure 2b, the recovery rate could reach 89.3% at 25 min. With a further extension of the reaction time, the recovery rate did not increase significantly, indicating that 25 min incubation time could meet the requirement for IMBs’ enrichment. Therefore, 25 min was chosen as the suitable incubation time.

#### 2.1.3. Optimization of Washing Solutions

In the oil–water mixture system, the IMBs would be unavoidably polluted by grease molecules during the specific binding with the target. Grease molecules would bind to the non-polar part of the antibody or the target, disturbing the specific binding of the antibody to the antigen and affecting the detection results, meaning that the washing solution is another key factor affecting the recovery rate. The experiment investigated the effect of PBS and 0.5% PBST solution (PBS solution containing 0.5% Tween 20) as a washing solution on the recovery rate, and the recovery rates were 76.5% and 89.3%, respectively. As illustrated in Figure 2c, there was a significant difference in the results between the two groups (*p* < 0.01), indicating that the appropriate dosage of Tween 20 had a significant effect on improving the recovery rate. As shown in the UPLC chromatogram in Figure 2d, no obvious interference peak appeared in corn oil samples, indicating the good purification result. As further increasing the concentration of Tween 20 in the PBS solution might cause interference peaks that affect the chromatogram results, 0.5% PBST solution was chosen as the suitable washing solution.

#### 2.1.4. Research on the Mechanism of Enrichment Effects

In order to explore the mechanism of enrichment effects, the quality control materials of corn oils were mixed with PBS solution and 2.5% PBST solution, extracted using a vortex oscillator, and then centrifuged to obtain the extraction solution to filter for detection by UPLC. The result showed that the extraction efficiency of PBS solution for ZEN was below the limit of detection (LOD), while it was 40.2% for 2.5% PBST solution. ZEN molecules had a weak polarity and strong lipophilicity, and were almost insoluble in PBS solution. Utilizing the solubilization effect of surfactants, the solubility of ZEN in aqueous phase was increased. Due to the low critical micelle concentration, nonionic surfactants were prone to forming micelles, showing excellent solubilization effects. In this test, when 2.5% Tween 20 was dissolved in PBS solution, about 40% of ZEN could be extracted in PBST solution, indicating the excellent solubilization effects of Tween 20. In addition, the addition of Tween 20 to the system improved the dispersibility of the oil phase in the aqueous phase, increased the contact area between the oil phase and the aqueous phase, and enabled the target toxin to quickly transfer between the oil phase and the aqueous phase. The essence of this reaction system was consistent with the DLLME principle, and Tween 20 and 2.5% PBST solution acted as a dispersant and extractant, respectively.

As we all know, under a certain temperature and pressure, if a substance was dissolved in two immiscible liquids that coexisted, the concentration ratio of the substance in the two liquids would be equal to a constant when it reached equilibrium. In this test, the distribution ratio of ZEN in the aqueous phase (2.5% PBST solution) and oil phase was 4:6. The IMBs, corn oil and reaction solution (2.5% PBST solution) were fully shaken and mixed by the mycotoxin automatic purification instrument. During the shaking and mixing process, ZEN was dissolved into the aqueous phase. As the IMBs constantly captured the dissolved ZEN in the aqueous phase, ZEN in the aqueous phase was always in an unsaturated state, and the ZEN concentration in the aqueous phase and oil phase could not achieve equilibrium, meaning that the ZEN in the corn oil was ceaselessly dispersed into the aqueous phase to form a dynamic equilibration. Utilizing this dynamic equilibration, the enrichment of ZEN by IMBs was realized.

### 2.2. The Selectivity and Specificity of the IMBs

**Figure 3:**
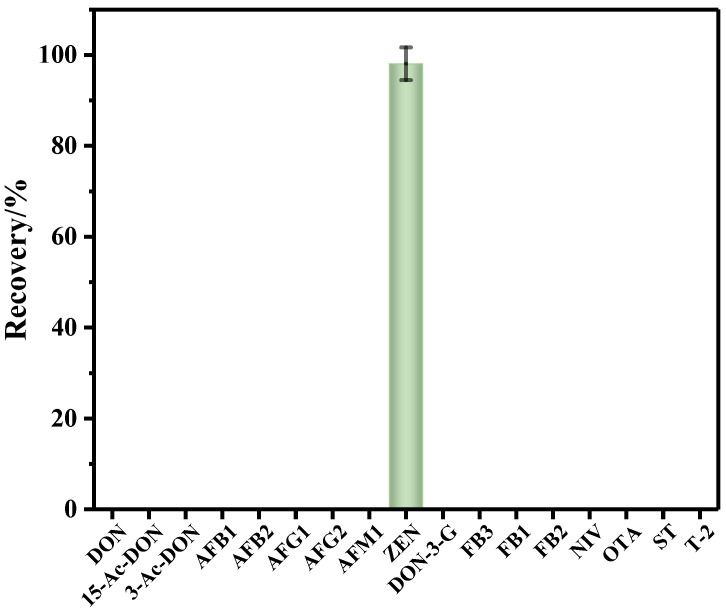
The recovery rate of the 17 kinds of mycotoxins pretreated with anti-ZEN IMBs, error bars represent SD.

The selectivity and specificity of the anti-ZEN IMBs to ZEN was evaluated by contrasting the recovery rate of ZEN and popular mycotoxins (i.e., aflatoxin B_1_ (AFB_1_), aflatoxin M_1_ (AFM_1_), aflatoxin B_2_ (AFB_2_), aflatoxin G_1_ (AFG_1_), aflatoxin G_2_ (AFG_2_), fumonisin B_1_ (FB_1_), fumonisin B_2_ (FB_2_), fumonisin B_3_ (FB_3_), nivalenol (NIV), ochratoxin A (OTA), sterigmatocystin (ST), T-2 toxin (T-2), deoxynivalenol (DON), 3-acetyl-deoxynivalenol (3-ADON), 15-acetyl-deoxynivalenol (15-ADON) and deoxynivalenol-3-glucoside (DON-3-G)). Blank corn oil samples spiked with ZEN and popular mycotoxins reference solutions (i.e., ZEN: 20.00 μg/mL, AFB_1_: 2.00 μg/mL, AFB_2_: 0.50 μg/mL, AFG_1_: 2.00 μg/mL, AFG_2_: 0.50 μg/mL, AFM_1_: 0.50 μg/mL, FB_1_: 20.04 μg/mL, FB_2_: 10.06 μg/mL, FB_3_: 10.20 μg/mL, NIV: 200.40 μg/mL OTA: 1.90 μg/mL, ST: 2.13 μg/mL, T-2: 2.00 μg/mL, DON: 151.05 μg/mL, 3-ADON: 40.16 μg/mL, 15-ADON: 20.00 μg/mL, DON-3-G: 25.10 μg/mL) were pretreated with the IMBs-DLLME method, and then subjected to detection. As shown in Figure 3, the recovery rate of ZEN was 98.1%, while the other mycotoxins’ toxins were below LOD, indicating the excellent selectivity and specificity of the anti-ZEN IMBs.

### 2.3. Method Validation

#### 2.3.1. Linearity and Sensitivity

**Figure 4:**
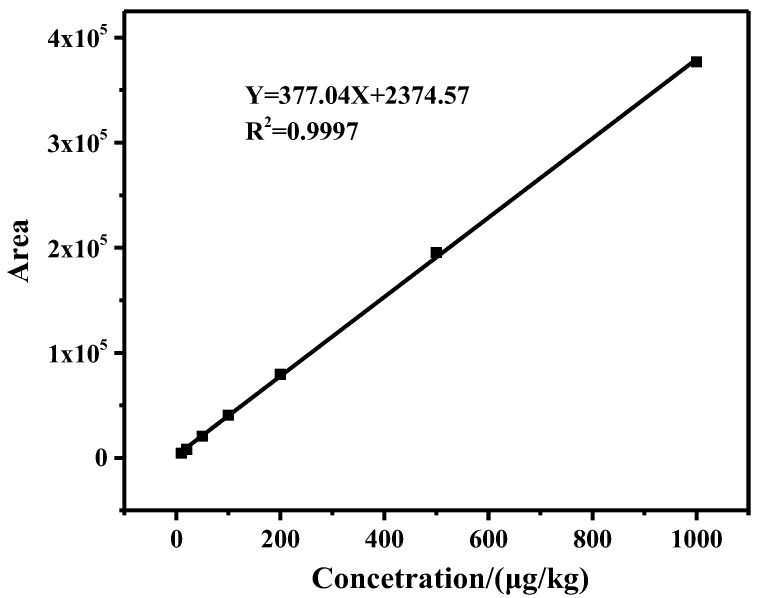
The standard curve of ZEN.

Under optimized experimental conditions, a series of standard solutions in the range of 10–1000 μg/kg were prepared and detected, and the calibration curve was established. By plotting the standard curve of peak area (Y) and standard solution concentration (X), the linear regression equation Y = 377.04X + 2374.57 was obtained, and the coefficient of determination (*R^2^*) was 0.9997 (Figure 4), which met the requirement of GB/T 27417-2017 for the linearity of the method (*R^2^* ≥ 0.99 for quantitative methods). The regression equation showed a strong linear correlation in the range of 10–1000 μg/kg. LOD and the limit of quantitation (LOQ) were obtained from the minimum concentration of ZEN that displayed a chromatographic peak area with signal to noise ratio (S/N) of 3 and 10, and the LOD and LOQ were 3.3 μg/kg and 10 μg/kg, respectively.

#### 2.3.2. Accuracy

To further assess this IMBs-DLLME pretreatment method, blank corn oil samples spiked with 142, 237, and 474 μg/kg ZEN reference solutions were pretreated with the IMBs-DLLME pretreatment method and then detected. The detection results of the recovery rate and relative standard deviation (RSD) are shown in Table 1. The IMBs-DLLME pretreatment method with UPLC-FLD detection showed satisfactory results; the recovery rate was in the range of 85.7–89.0%, and the RSD was in the range of 1.8–2.9%, demonstrating excellent trueness and precision, which met the standard Commission Regulation (EC) No 401/2006 [29].

### 2.4. Method Comparision

**Figure 5:**
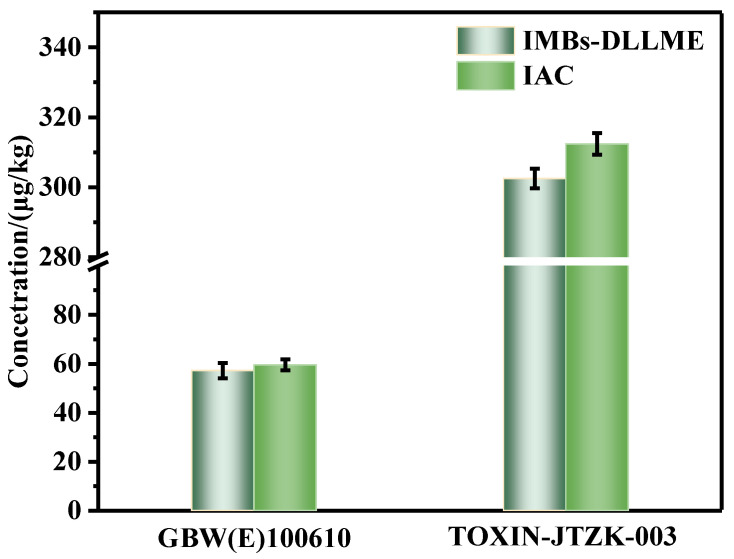
The detection results of the IMBs-DLLME pretreatment method and the IAC pretreatment method for corn oil samples; error bars represent SD.

To obtain an objective evaluation of the proposed IMBs-DLLME pretreatment method, it was compared with the traditional IAC pretreatment method. The national certified standard substance GBW(E)100610 (60 ± 5.1 μg/kg) of ZEN in corn oil matrix and the quality control materials TOXIN-JTZK-003 (320 μg/kg) were applied for verification. The two pretreatment methods were conducted, and UPLC-FLD detection was carried out. The detection results obtained by the two pretreatment methods was shown in Figure 5. Significance analysis was conducted for the two groups of data and *p* > 0.05, indicating that the two pretreatment methods showed a good agreement without significant differences, and the proposed pretreatment method had good accuracy. The proposed IMBs-DLLME pretreatment method amalgamated extraction and purification steps into one step, eliminating the pre-extraction with the organic reagents step. The whole process required about 43 min and 10–24 samples could be pretreated simultaneously, while the IAC pretreatment method required extraction, centrifugation and IAC purification steps, and the whole process took about 127 min. In addition, the proposed pretreatment method also had obvious advantages in the dosage of organic reagents, and only 0.5 mL organic reagent was required to avoid the subsequent production of organic waste liquid, while the IAC pretreatment method required at least 10 mL organic reagents in the extraction step (Table S1). Furthermore, the common rapid detection methods also required at least 10 mL organic reagent for extraction, and then used a little extraction solution for detection [30,31]. These methods could cause the subsequent production of organic waste liquid, especially for batch sample detection, and the environmental pollution could not be ignored. Therefore, the proposed IMBs-DLLME pretreatment method exhibited obvious advantages in terms of the consumed time, the degree of automation and the dosage of organic reagents, and possessed the characteristics of automation, high efficiency and environmental protection.

### 2.5. Application in Real Samples

**Figure 6:**
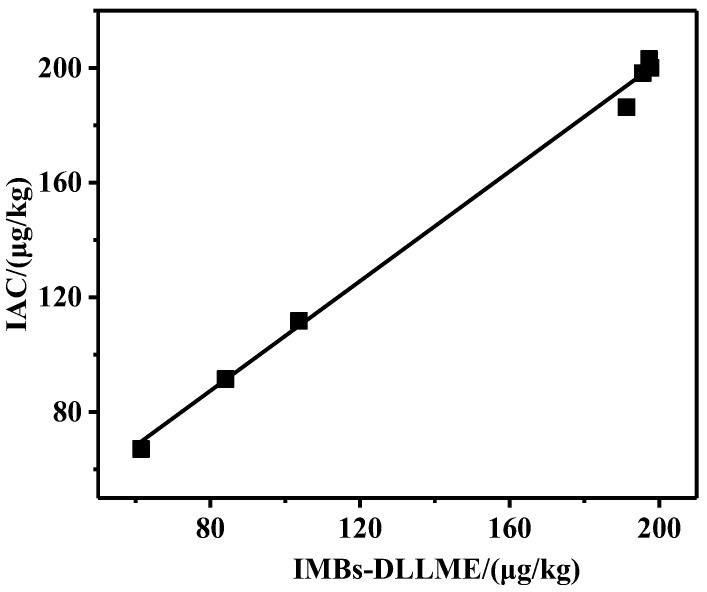
Comparison of detection results for real samples.

In order to validate its application in real samples, the proposed IMBs-DLLME pretreatment method and the IAC pretreatment method were conducted to pretreat seven samples purchased from supermarkets, and then UPLC detection was performed (Table S2). The results showed that ZEN was detected in all samples with the concentration of 60–200 μg/kg, indicating that the pollution of ZEN in corn oil could not be ignored. The results’ analysis is shown in Figure 6. A positive correlation between the two group results was obtained, with a slope of 0.956 and a *R^2^* of 0.997, indicating that the results from IMBs-DLLME method agreed well with that of the IAC method. Therefore, it could be considered that the pretreatment effects of these two pretreatment methods were consistent, and the developed pretreatment method was a considerable alternative to the IAC method.

## 3. Conclusions

In conclusion, the automatic IMBs-DLLME pretreatment method of ZEN in corn oil, which skillfully combined the advantages of IMBs and DLLME technology, was established, and the quantitative detection and analysis was carried out with UPLC-FLD. The reaction solution, incubation time, and washing solution were optimized, and the mechanism of the enrichment effects was studied. Under the optimal conditions, the poposed method had good linearity, LOD, LOQ, accuracy and precision. Compared with the traditional IAC pretreatment method, this method integrated the extraction and purification steps. A certain volume of corn oil could be directly added into the reaction well to realize the automatic purification process, greatly shortening the consumed time and reducing the operation steps. In addition, the addition of surfactant effectively solved the problem of non-polar molecular solubility in aqueous solutions. The application of surfactant formed micelles increased the solubility of weak-polarity ZEN molecules in aqueous solutions, helping to enrich the IMBs. As a new high-throughput technology for the automatic pretreatment of ZEN in corn oil, the proposed IMBs-DLLME method was efficient and environmentally friendly, and could be applied as an alternative to the traditional IAC method. Furthermore, the developed method provided a reference for the pretreatment of other targets, provided technical support for improving the detection and monitoring ability of mycotoxins, and possessed excellent promotion prospects and application value.

## 4. Materials and Methods

### 4.1. Chemicals and Materials

HPLC-grade methanol was purchased from Fisher Scientific (Suwanee, GA, USA). Ultra-pure water was purchased from Watsons (Beijing, China). Buffer solution salt (PBS) pack and 2-morpholinoethanesulphonic acid (MES) buffer solution were purchased from Sigma Aldrich (Shanghai, China) Trading Co., Ltd. Tween 20 surfactant was purchased from Guangfu Chemical Research Institute (Tianjin, China). Acetonitrile was purchased from Shanghai Aladdin Biochemical Technology Co., Ltd. (Shanghai, China). Glycine was purchased Sangon Biotech Co., Ltd. (Shanghai, China). NHS group activated agarose magnetic beads (NHS-MBs) were purchased from Beaver Bioscience Inc. (Jiangsu, China). Monoclonal antibody of ZEN was purchased from Chuangpu Biotechnology Co., Ltd. (Wuxi, China). Certified reference materials of corn oil GBW(E)100610, quality control materials of corn oil TOXIN-JTZK-003 and standard solutions of ZEN in methanol GBW(E)100301 were purchased from Academy of National Food and Strategic Reserves Administration (ASAG) (Beijing, China). ZEN standard solutions were prepared by dilution with 90% methanol/H_2_O solution, and then placed in vials at 4 °C for use. ZEN and popular mycotoxins standard solutions were purchased from Romer Labs, Inc. (Union City, MO, USA).

### 4.2. Instruments and Equipment

The Waters ACQUITY UPLC H-class system (Waters, Manchester, UK); Mycotoxin automatic purification instrument (JJHZ10, Beijing Dongfu Jiuheng Instrument Technology Co., Ltd., Beijing, China); multi-tube vortex mixer (MTV-100, Hangzhou Aosheng Instrument Co., Ltd., Beijing, China); desktop high-speed freezing centrifuge (5810R, Eppendorf, Germany); Quick solvent dryer (N-EVAP112, Organomation, Berlin, MA, USA).

### 4.3. UPLC-FLD Conditions

The chromatographic column is Waters BEH-C18 column (100 mm × 2.1 mm, 1.7 μm); The column temperature and sample temperature are 40 °C and 10 °C, respectively; the mobile phase is H_2_O/acetonitrile, with a volume ratio of 45:55 and a flow rate of 0.2 mL/min; Injection volume is 10 μL; fluorescence detection wavelength: excitation wavelength 303 nm, emission wavelength 440 nm; data were processed and analyzed using Waters EMPOWER3 software.

### 4.4. Synthesis and Evaluation of Anti-ZEN IMBs

Anti-ZEN IMBs was synthetized from NHS-MBs, and the synthesis steps were as follows: at first, the NHS-MBs solution was mixed well, and 100 mL of the solution was taken; thenm a magnetic separator was used to obtain the solid, and the solid quickly dispersed into the absolute ethanol solution. This was stirred for 10–15 s; then, a magnetic separator was used to obtain the solid for standby. Then, 16.4 mL ZEN antibody (24.38 mg/mL, purity 84%) was dispersed to the MES buffer solution (pH = 6.0), and then added to the above solid and incubated for 2.5 h under room-temperature fluctuation blending. After incubation, the solution was magnetically separated to obtain the solid, and then the solid was dispersed to 100 mL MES solution containing 2% glycine to block non-specific adsorption sites for 2.5 h. After blocking, the solution was magnetically separated to obtain the solid. The solid was rinsed with 0.1% PBST solution and PBS solution three times. Finally, the solid was added to 200 mL PBS solution and stored at 4 °C. To verify the maximum specific adsorption of anti-ZEN IMBs and exclude the non-specific adsorption of NHS-MBs, 100 μL anti-ZEN IMBs solution was taken and mixed with ZEN standard solution, and subjected to UPLC detection. As a comparison test, 100 μL NHS-MBs solution was processed by the same steps. The maximum specific adsorption of 100 μL IMBs solution was 145.2 ng ZEN, and that of NHS-MBs solution was below LOD.

### 4.5. Automatic Purification Process

The automatic purification procedure of mycotoxin automatic purification instrument is as follows: 200 μL IMBs solution, 250 μL corn oil and 300–600 μL reaction solution are mixed in the sample well for 25 min, and magnetic absorption is performed for 6 min; IMBs are transferred to the washing well (4 in all), each well is washed for 1 min, and magnetic absorption occurs for 1 min. The IMBs are transferred to the elution well, eluted for 1 min, and undergo magnetic absorption for 1 min. After that, the IMBs are transferred to the recycling well, and the purification process is completed. The entire automatic purification process takes approximately 43 min, and 24 samples could be pretreated at the same time.

### 4.6. Sample Extraction and Manual IAC Purification Process

The IAC pretreatment processes were conducted on the basis of the national standard (GB5009.209-2016) with some modifications. A total of 2 g corn oil and 10 mL 90% acetonitrile/H_2_O were put into the centrifuge tube, and the solution was vortexed for 20 min for ZEN extraction. After vortexing, the solution was centrifuged to obtain extraction solution. Then, 2 mL extraction solution was diluted with 18 mL 0.1% PBST solution. A total of 20 mL diluted solution flowed through ZEN IAC with 1–2 drops per second, and then was rinsed by 10 mL PBS solution. 2 mL HPLC grade methanol was applied to elute ZEN from IAC. The eluent was blowed under N_2_ stream at 50 °C and reconstituted with 1 mL 50% methanol/H_2_O and then UPLC detection was conducted.

### 4.7. Validation of Analytical Method

The IMBs-DLLME pretreatment method with UPLC detection method was assessed by methodology validation such as recovery rate, accuracy, precision, linear range, LOD, and limit of quantitation (LOQ). The number of independent repetitions was 3 (*n* = 3). Recovery rate, accuracy and precision were appraised with blank corn oil samples spiked with with 142, 237, and 474 μg/kg ZEN standard solution. To ensure the credibility of the test, the spiked samples were mixed well, and the container was opened for 8 h at room temperature to evaporate solvent from the samples. The concentration level of the spiked sample was repeated to evaluate the repeatability of the method. Linear range, LOD and LOQ were obtained by the detection of standard solutions with different concentrations. The detection results of the proposed IMBs-DLLME method were taken as X, those from the IAC method as Y, and the slope and *R^2^* were obtained by plotting the curves.

## Figures and Tables

**Table 1:** Spiked recovery rate and RSD (*n* = 3).

Toxins	Concentration (μg/kg)	Recovery Rate	RSD
ZEN	142	87.5%	1.8%
	237	89.0%	2.9%
	474	85.7%	2.7%

## Data Availability

Data available on request from the authors.

## Supplementary Materials

The following supporting information can be downloaded at: https://www.mdpi.com/article/10.3390/toxins15050337/s1, Table S1: Comparison of the proposed IMBs-DLLME pretreatment method with the IAC pretreatment method. Table S2: Detection results of real samples.
