# Supplementary material for: Automatic Pretreatment of Dispersive Liquid Liquid Microextraction Based on Immunomagnetic Beads Coupled with UPLC-FLD for the Determination of Zearalenone in Corn Oils
Source: Toxins (Basel). 2023 May 15;15(5):337. doi: 10.3390/toxins15050337 (PMC10221060; doi:10.3390/toxins15050337)
Supplement: Supplementary file 1 [file toxins-15-00337-s001.zip › toxins-2370954-supplementary.pdf]

toxins

MDPI

Table S1. Comparison of the proposed IMBs-DLLME pretreatment method and the traditional IAC pretreatment method.

Process	Vortex	Centrifugation	Clean-Up	Dilution	Nitrogen evaporation	Total
IAC	20 min [a] /10 mL [b]	5 min	42 min /2 mL	no need	60 min	127 min /12 mL
IMBs-DLLME	no need	no need	43 min /0.5 mL	2 min	no need	45 min /0.5 mL

[a] The time consumed in this process. [b] The organic reagents consumed in this process.

Table S2. Detection results of real samples.

Sample	IAC (μg/kg)	IMBs-DLLME (μg/kg)
#1	84.1	91.5
#2	195.6	198.2
#3	197.7	200.1
#4	197.3	203.1
#5	191.3	186.3
#6	103.7	111.8
#7	61.5	67.1
